# A Proposal for an Intermediate Care Unit-Quality Measurement Framework

**DOI:** 10.1155/2018/4560718

**Published:** 2018-07-29

**Authors:** Joost D. J. Plate, Linda M. Peelen, Luke P. H. Leenen, Roderick M. Houwert, Falco Hietbrink

**Affiliations:** ^1^Division of Surgery, University Medical Centre Utrecht, Utrecht, Netherlands; ^2^Julius Center for Health Sciences and Primary Care, University Medical Center Utrecht, Utrecht, Netherlands; ^3^Departments of Anesthesiology and Intensive Care Medicine, University Medical Center Utrecht, Utrecht, Netherlands

## Abstract

*Rationale*, *Aims*, *and Objectives*. The Intermediate Care Unit (IMCU) is a hospital unit which is logistically situated between the hospital ward and the Intensive Care Unit (ICU). There is debate regarding the value of the IMCU. Understanding its value is compromised by the lack of adequate quality indicators. Therefore, this study identifies currently used IMCU indicators and evaluates their usefulness. *Methods*. Through a systematic literature search, currently used quality indicators were identified and evaluated for their importance using a proposed IMCU-specific quality measurement framework. *Results*. From 4034 titles and abstracts, 168 articles were selected for full-text review. Of these, 22 articles were included, which reported IMCU quality at the level of the IMCU (*n* = 12), the ICU (*n* = 5), both IMCU and ICU (*n* = 3) or hospital level (*n* = 2). At the IMCU, the IMCU mortality (*n* = 16), discharge-to-ICU rate (*n* = 7), in-hospital IMCU mortality (*n* = 7), and length of stay (*n* = 6) were most frequently reported. Three studies compared the effect of different structures of the IMCU on its utilization or hospital outcome. *Conclusions*. Current focus in IMCU quality research is towards measuring quality at the IMCU itself. Since the influence of the structure of IMCUs on its utilization and its effects on hospital outcome are only rarely investigated, attention should shift towards these important issues in further research. The proposed IMCU quality measurement framework can thereby serve as a helpful tool.

## 1. Introduction

The Intermediate Care Unit (IMCU), Step-Down Unit or High-Dependency Unit, is a hospital unit which is logistically situated between the hospital ward and the Intensive Care Unit (ICU) [1]. The IMCU has emerged as an alternative to the ICU to provide supportive critical care for severely ill patients, and reduce the pressure on costly ICU beds [2]. However, there is debate regarding the value of the IMCU [3–5]. Understanding the value of IMCUs is compromised by its large heterogeneity, as IMCUs can be stand-alone or integrated into the ICU, and can admit very different patients and case-mixes. An approach to understand and compare outcomes of differently formatted IMCUs is through severity of illness measurements [6, 7]. However, the comparison of organizational structures such as the IMCU is much more complex due to its influence on other hospital units, such as the hospital ward and ICU [8].

At the ICU, there are consensus definitions on meaningful quality indicators [9]. At the IMCU, there is no earlier research performed in this field. And although the IMCU historically relies heavily upon ICU literature [5], it is unlikely that all ICU quality measures can be applied one-on-one to the IMCU as the IMCU is not a high-end critical care facility and thus has limited supportive options; that is, deteriorating patients at the IMCU should timely be transferred to the ICU for maximum supportive care.

As a first step in the process towards a consensus of IMCU quality indicator definitions, we identified currently used quality indicators for the IMCU. Subsequently, these were evaluated for their usefulness through the application of a proposed IMCU-specific quality measurement framework. This aims to support and provide direction for future research towards qualitative assessment and comparison of IMCUs.

## 2. Materials and Methods

### 2.1. Information Sources

To investigate which quality indicators are currently used in the IMCU literature, a comprehensive literature search was performed in multiple electronic databases (Medline, Embase, and Cochrane). All publications up to 10.09.2016 were searched. The conducted title/abstract search was broad and similar to a previous report [1]. The following keywords were used: “Medium Care Unit” OR “Intermediate Care Unit” OR “High Care Unit” OR “High Dependency Unit” OR “Progressive Care Unit” OR “Step Up Unit” OR “Step Down Unit” OR “Transitional Care Unit” and synonyms of those (see Supplementary file 2 for all search terms). No Mesh terms were available. A cross-reference check of all articles in full-text review was performed.

### 2.2. Study Selection

As inclusion criteria for full-text review and data abstraction, the following terms were used: (1) published in English or Dutch and (2) reporting quality indicators of an IMCU or of an ICU with IMCU. As for quality indicators, all articles were included that provided any information about the quality of the IMCU directly or indirectly by analysis of ICU or hospital outcomes.

Excluded were articles about cardiac, obstetric, gynaecologic, paediatric, and psychiatric care units due to their specific small spectrum model of care for well-defined disease entities. This stands in contrast to the possibility to harbour different patient groups and diseases in IMCUs such as surgical, general medical, or neurological IMCUs, for which the assessment of quality is potentially different. Articles were excluded if they described a transitional care unit between hospital and nursing homes, since these were not comparable to the IMCUs logistically situated between ICU and ward. Articles describing the outcome of a specific disease or treatment at the IMCU were also excluded, since these disease-specific studies investigated specific outcome of a disease among IMCU admissions. This was thought not to adequately reflect the overall quality. Also excluded were case reports, conference abstracts, and reviews as information concerning outcome parameters were deemed not detailed enough.

### 2.3. Data Extraction

All reported quality indicators were extracted from included studies. As the purpose of this article was descriptive and no existing tool for risk of bias assessment was available for this type of systematic review on observational studies, a formal risk of bias assessment was not conducted.

### 2.4. Proposed IMCU-Specific Quality Measurement Framework

After identification of the quality indicators, an IMCU quality measurement framework was proposed using 3 general approaches to the assessment of quality of care, as proposed by Donabedian [10]. Structure (A), such as the available equipment and nurse-to-patient ratio, refers to the aspects of the settings in which care occurs. Process (B), such as the interventions performed at the IMCU or the type of admissions in terms of case-mix severity, entails what is done in giving and receiving care. Outcome (C), such as mortality, is the effect of care on the individual or population health care status. The underlying hypothesis to this approach is that a good structure leads to a good process which in turn leads to a desired outcome. With knowledge of these relationships, the structure (and processes) can be adjusted or used to improve or assess patient outcomes, which are the eventual parameters of interest.

Examples of IMCU structure are its facilities (e.g., availability of vasopressors or high-flow nasal cannula oxygen therapy), its location (stand-alone or integrated into the ICU), and organization (management format: intensivist in charge (closed) or specialist in charge (open)). Examples of IMCU process are the type of admissions (e.g., postoperative or emergency trauma admissions) and ICU accessibility (e.g., number of refused ICU admissions, assessed as having an ICU indication). As an example of the utilization of the framework, lowering the nurse-to-patient ratio (the structure) may lower the severity of illness of admitted patients (the process). Or, if a different process is desired (e.g., more severely injured patients), the structure can be changed to reach this goal (e.g., a higher nurse-to-patient ratio or the availability of high-flow nasal cannula oxygen therapy at the IMCU).

For the proposed IMCU-specific quality measurement framework, these approaches were combined with the location of measurement of the IMCU quality: at the IMCU itself, at the ICU, or at the hospital level. The rationale behind this is that the IMCU directly affects both the ICU and the hospital (ward) and vice versa, and thus the qualitative performance (and with it, the quality assessment) of the IMCU cannot be regarded as a separate entity. As an example, a well-performing ICU with readily accessible ICU beds likely decreases the mortality at the IMCU through the rapid possibility of discharging deteriorating patients to the ICU. Possibly, this also decreases hospital-wide mortality. In addition, research towards the quality of implementing IMCUs typically focusses on the effects of the IMCU on the ICU as there is a risk of receiving more deteriorated patients from the IMCU at the ICU [11, 12].

**Figure 1:**
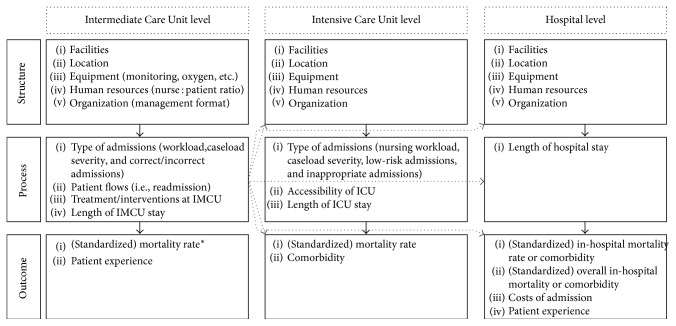
The Proposed Intermediate Care Unit-Quality Measurement Framework. This figure shows—on the left side—the different approaches to the assessment of the quality of care, as described by Donabedian [10]. It also shows—on the top side—the geographical location (level) at which these are measured. The solid arrows show relationships between the approaches to assessment of the quality of care, while the dashed arrows depict the relationships between quality categories on different geographical locations. From this framework, it follows that quality measurement of the IMCU is closely related to other parts of the hospital.

This proposed IMCU-specific quality measurement framework is shown in Figure 1. The rows denote the three approaches to quality measurement (structure, process, and outcome), whereas the columns indicate the location where this quality indicator is being measured (at the IMCU, at the ICU, or at the hospital level). This framework can be used to visualize and clarify the possible effects of IMCU performance on forwarded units. To further explain the interpretation of this framework, a hypothetical example is briefly discussed here and elaborated in more detail in Supplementary file 1.

Let us say that, as a fictive example, the IMCU in a certain hospital decreases its nurse-to-patient ratio (IMCU structure). This likely affects the process at the IMCU by admitting less severe patients, which leads to a lower readmission rate, a lower discharge-to-ICU rate, a decreased length of stay (IMCU process), and a decreased mortality (IMCU outcome). In turn, this change in IMCU process may lead to more beds necessary at the ICU (ICU structure), a lower case-mix severity at the ICU, less accessibility of the ICU (ICU process), and a lower ICU mortality rate (ICU outcome). Potentially, due to less optimal allocation of available resources, the hospital then needs, overall, more nurses (hospital structure), the in-hospital length of stay increases (hospital process), and the overall mortality and costs may increase (hospital outcome). Of note is that this is purely a hypothetical example of a possible application of this IMCU-specific quality measurement framework and is not based on empiric evidence.

### 2.5. Data Synthesis and Analysis

The values of the reported quality indicators were analysed by calculating the range of each indicator. This quantitative analysis was performed on a study level including all articles, with the following exceptions: if multiple studies described quality indicators at the same unit, the most recent article was used; if studies described multiple IMCUs without providing unit-specific data, they were included as one study in the analyses; on the other hand, if an article described more than one unit, namely, before and after changing its structure, both units (time periods) were included.

Clinical heterogeneity between IMCUs with respect to their characteristics turned out to be too large to warrant pooling of study findings; hence, a meta-analysis could not be conducted.

## 3. Results

### 3.1. Study Selection

**Figure 2:**
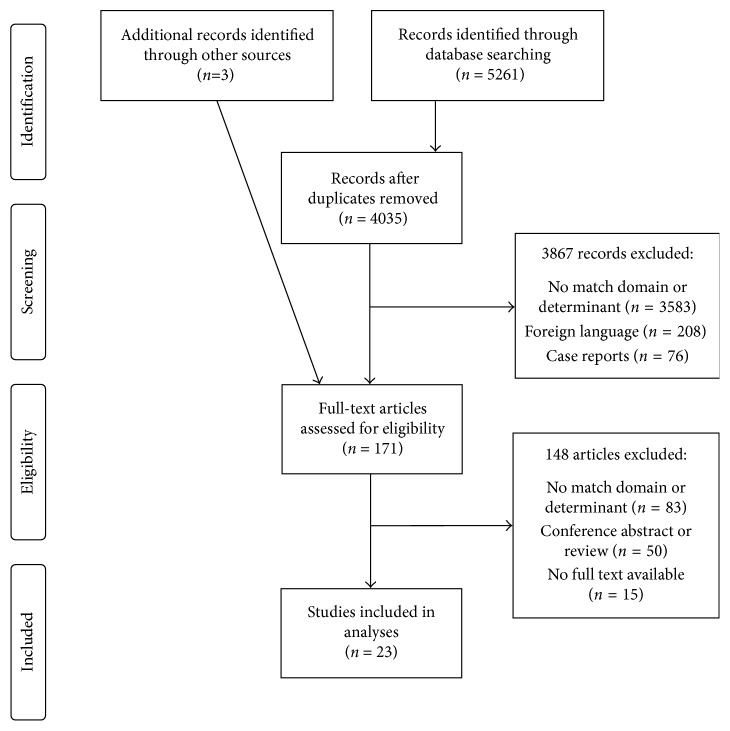
Preferred Reporting Items for Systematic Reviews and Meta-Analyses (PRISMA) flow diagram for study selection [13].

From 4037 titles and abstracts, 171 articles were selected for full-text review (Figure 2), of which three were found via cross-reference checking. Of these, 23 studies were included. These described the performance of 22 units, since one unit's performance was described by two articles [6, 7]. Two studies described their IMCU before and after changes in management or location structure and were therefore each included twice in the quantitative analysis [14, 15]. Thus, a total of 24 IMCUs were covered in the quantitative analysis.

### 3.2. Study Characteristics

All included studies were cohort studies, using either an IMCU cohort (*n* = 12) [7, 16–26] or ICU cohort in presence and absence of the IMCU (*n* = 5) [11, 12, 15, 27, 28] or using both IMCU and ICU cohorts (*n* = 3) [29–31]. Two studies used a hospital cohort to compare the overall in-hospital mortality before and after introduction of the IMCU [2, 22]. Ten studies were descriptive and noncomparative with respect to the IMCU structure [7, 16–18, 20, 21, 23–26], seven studies performed a before and after introduction comparison including an IMCU (and ICU) cohort [2, 14, 19, 22, 29–31], and five studies compared their ICU cohorts with and without presence of an IMCU in the hospital [11, 12, 15, 27, 28]. An overview of all included studies with their study characteristics is provided in Supplementary file 3.

### 3.3. Identified Quality Indicators and Their Position in the IMCU-Specific Quality Measurement Framework

**Figure 3:**
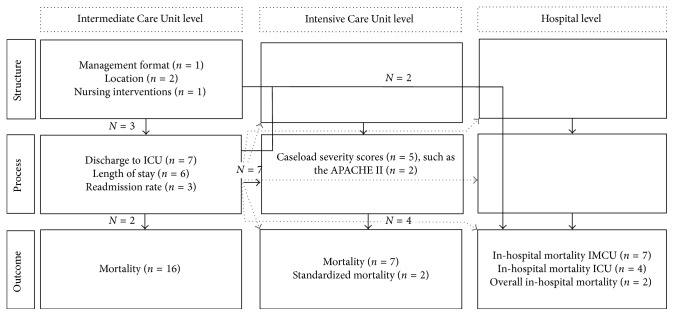
The Currently Reported Quality Indicators and their position in the Proposed Intermediate Care Unit-Quality Measurement Framework. In this figure, the numbers in the boxes represent the frequencies of reporting of quality indicators and their position in the proposed intermediate care unit-quality measurement framework (Figure 1). The numbers in the grey boxes represent the numbers of comparative studies reporting on the relationship (the black arrows) between boxes and reported how a change in one box affected the other one.

Figure 3 describes the position of all quality indicators in the proposed quality measurement framework. The most frequently reported quality indicators were the mortality at the IMCU (*n* = 16) [2, 6, 7, 14, 16–26, 29], the discharge-to-ICU rate (*n* = 7) [6, 7, 14, 20–23], the in-hospital mortality of IMCU patients (*n* = 7) [2, 12, 14, 16, 17, 19, 22], and ICU mortality (*n* = 7) [2, 11, 12, 22, 27–29].

Nine studies (of which 7 at the ICU level) related between elements of the framework: the relationship between the IMCU structure, process, and hospital outcome (*n* = 3) [14, 19, 31] or between IMCU process and ICU process and outcomes (*n* = 7) [2, 11, 12, 15, 27–29].

### 3.4. Summarized Quality Indicators

Table 1 provides an overview of the ranges of reported quality indicators. This table shows that the IMCU mortality (1.2%–19.0%), discharge-to-ICU rate (1.6%–10.0%), and in-hospital mortality of IMCU patients (8.1%–19.7%) varied widely. In presence of the IMCU, the ICU case-mix severity (APACHE III of 14.2–49.6 versus 13.40–34.50), ICU readmission rate (5.8%–15.8% versus 5.0%–9.0%), and ICU mortality rate (7.3%–23.0% versus 1.1%–16.6%) were higher [11, 12, 27–29]. In one study, the ICU mortality rate was lower (35.5% versus 40.1%) in presence of the IMCU [2].

The higher ICU mortality was adjusted for the increased case-mix severity in two studies, with conflicting results. One found an increased ratio of observed to predicted in-hospital mortality of ICU patients after introducing an IMCU (0.83 (95% CI 0.66–1.03) before versus 1.24 (95% CI 1.05–1.46) after introduction of the IMCU) [27]. Another multicentre study found a lower in-hospital ICU mortality (OR 0.63 with 95% CI 0.45 to 0.88) [12]. This lower mortality was only present in the more critically ill patients who required intensive care treatment at the ICU (OR 0.54 with 95% CI 0.37 to 0.80) and not in those admitted for merely observation (OR 1.15 with 95% CI 0.65 to 2.03).

## 4. Discussion

There is no standardized format to report quality assessment of IMCU functioning, which hampers comparison of data between reports and the logistic intermediate nature of these units thwarts the interpretation of outcome measures. This study provides a structured overview of the different quality indicators currently used. Furthermore, it proposes an IMCU-specific quality measurement framework. It follows that there is a close relationship between the structure and process of delivering care at the IMCU and the other parts of the hospital, ideally warranting a hospital-outcome-based approach, that is, on in-hospital mortality. Currently, however, the focus is largely descriptive and focussed on patient-based IMCU outcomes, such as mortality at the IMCU. To allow for comparison, outcome measures should be adjusted for the severity of illness (i.e., with the IMCUSS), the type of admissions (e.g., postoperative admissions), or the level of required supportive care (i.e., through nursing intervention systems).

The rationale for the use of the proposed quality measurement framework is that all three locations of measurements are of considerable importance in assessing the quality of the IMCU. Combined with the three approaches to quality, this forms a complex though necessary framework linking the quality of the IMCU to the other parts of the hospital. Of special importance is therefore the relationship between the different components of this framework.

Currently, IMCU process and IMCU outcome quality measurements are most frequently reported. In reporting the ranges, this heterogeneity is also observed. As an example, the actual average mortality at the IMCU per unit ranged from 1.2% to 19.0%. This is potentially—among others—explained by differences in case-mix severity, nurse-to-patient ratio, utilization of the IMCU (purely step-down unit or also a step-up unit), hospital regulations, such as the use of vasoactive medications, and the training of the nurses and medical staff. It may also be explained by the inclusion of patients after a decision to forgo life-sustaining treatments, which distorts the mortality rates. Moreover, only one study used a time interval (ICU transfer <24 hours of admission) in their outcomes. A time interval is important to consider, as it can distinguish between inadequate triage at admission (e.g., ICU transfer <24 hours) and deterioration after admission.

The effects of the relationship between the components of the quality measurement framework are rarely studied. This is probably due to the descriptive nature of studies reporting on their IMCU, since only a few comparative studies reported the effect of the IMCU structure on patient flows at the IMCU itself and/or the effect of the IMCU structure on hospital outcomes (*n* = 2) [14, 19].

In the ideal situation, both the relationship between the IMCU structure and IMCU process and the relationship between the IMCU process and hospital outcomes are known. The first part can be measured on a daily basis at IMCUs and can be (directly) adjusted, while the latter part (the hospital outcome) is eventually of interest for the individual patient, the overall population, and the allocation of health care resources (costs). Within the hospital outcome group, it should be noted that the in-hospital mortality of IMCU patients is affected by other parts of the hospital, while the IMCU has its effect on the in-hospital mortality of ICU patients as well as the overall in-hospital mortality. Incorporated into these recommendations is the feasibility of data collection, as it is not feasible to collect data from all hospitalized patients in the hospital. This may, however, become less relevant with the increasing availability of electronic patient data.

Through analysing the relationships between IMCU structure and process and IMCU process on hospital outcomes, the optimal IMCU structure can be determined to achieve (hospital-desired) processes at the IMCU while maximizing patient outcomes and minimizing the costs of data collection. It is unlikely that all ICU quality measures can be applied one-on-one to the IMCU as the IMCU is not a high-end critical care facility and thus has limited supportive options; that is, deteriorating patients at the IMCU should timely be transferred to the ICU for maximum supportive care.

The strength of this study is that it provides a framework to categorize and structure the aspects of quality measurement of the IMCU, along with a structured overview of currently used quality indicators, although limited research has been done in this field. It thereby shows the current and desired focus of further research in this field. By providing this overview, further focussed research can be done towards the qualitative performance of IMCUs so that a thorough and valid comparison between IMCUs can be made in this disparate research field.

This study is probably limited by a high level of publication bias, since authors possibly only report about their IMCU if it is a successful unit. Thus, it could be that our study misses important quality indicators which were not reported. However, it was not the aim of our literature search to be as comprehensive as possible since this literature search was solely performed to—globally—reflect what is current practice in assessing the quality of IMCUs and therein complement the proposed quality measurement framework. Another limitation is that the desired focus of IMCU quality research is not based on an overall consensus, mainly since this was currently not considered feasible in this disparate research field. Additionally, the proposed framework is not validated nor standardized. Also, this study focussed on general IMCUs as opposed to specialized IMCUs (e.g., cardiac, obstetric, gynaecologic, and stroke units) and therefore the results do not necessarily apply to these other types of IMCUs. Further, this study included publications which were more than 20 years old and which therefore may not reflect current medical practice. Finally, only a limited number of (patient-reported) outcomes were reported in these articles.

## 5. Conclusions

Current focus in IMCU quality research is patient based and measures quality at the IMCU itself. However, since the influence of (1) the structure of IMCUs on its utilization and (2) its effects on hospital outcomes are only rarely investigated, attention should shift towards these important issues in further research. This way, the optimal IMCU structure can be determined to achieve (hospital-desired) IMCU processes while maximizing patient outcomes.

## Figures and Tables

**Table 1:** Identified reported quality indicators with their range of values and frequencies of peporting.

	Quality indicator	Range		Number of studies reporting (*N*)
Intermediate Care Unit level	Mortality (%)	1.2–19.0		16
	Discharge-to-ICU rate (%)	1.6–10.0		7
	Length of stay (days)	0.9–4.0		6
	Readmission rate (%)	3.8–6.3		3
	Discharge-to-ICU <24 hours rate (%)	6.1		1

		Without IMCU	With IMCU	
Intensive Care Unit level (difference with and without IMCU)^*∗*^	Mortality (%)	1.1–40.1	7.3–35.5	7
	Length of stay (days)	1.1–7.5	1.4–8.5	4
	Readmission rate (%)	5.0–9.0	5.8–15.8	3
	Caseload severity (APACHE III) [32]	13.4–34.5	14.2–49.6	4
	Inappropriate use of ICU beds: no active treatment (% of admission days)	3.2	0.01	1
	Inappropriate use of ICU beds: TISS-28 < 20 (% of admission days) [11]	18.7	9.7	1
	Low-risk monitor ICU admissions (%)	65.3	27.6	1
	Accessibility: refusal patients with ICU indication (%)	10.6	7.7	1

		Range		
Hospital level	In-hospital mortality IMCU patients (%)	8.1–19.7		7
	In-hospital length of stay IMCU patients (days)	16.3–38.0		3
		Without IMCU	With IMCU	
	In-hospital mortality ICU patients (%)	2.9–58.0	11.9–31.4	4
	In-hospital length of stay ICU patients (days)	11.0–26.5	13.9–37.3	2
	Overall in-hospital mortality (%)	2.2–4.5	3.2–3.9	2

This table shows the identified quality indicators at Intermediate Care level, Intensive Care level, and Hospital level. It also shows the range of values with the frequency of which each quality indicator was reported. The identified indicators frequently present are the IMCU (in-hospital) mortality, discharge-to-ICU rate, and the IMCU length of stay. The reported ranges are broad, indicating a large heterogeneity in IMCUs. Care should be taken not to ascribe a causal effect between the columns without the IMCU and with the IMCU, since no information regarding the difference per study can be extracted from this table. For more detailed information per included study, see Supplementary file 3. ^*∗*^The only study at measuring IMCU quality at ICU level did so comparing the situation before the implementation with after the implementation of the ICU. No different designs or formats of IMCUs were compared. IMCU =Intermediate Care Unit; ICU = Intensive Care Unit; APACHE III = Acute Physiology and Chronic Health Evaluation III; TISS-28 = Therapeutic Intervention Scoring System-28.

## Data Availability

The data used to support the findings of this study are included within the article.

## Abbreviations

IMCU: Intermediate Care Unit
ICU: Intensive Care Unit
APACHE III: Acute Physiology and Chronic Health Evaluation III.

## Supplementary Materials

Supplementary Materials Supplementary file 1: an example of the application of the proposed intermediate care unit-quality measurement framework. Supplementary file 2: the used search terms. Supplementary file 3: an overview of the included studies with a short description, the used comparison, severity of illness at IMCU or ICU level, and described outcomes on all three levels.
