# Supplementary material for: A Proposal for an Intermediate Care Unit-Quality Measurement Framework
Source: Crit Care Res Pract. 2018 Jul 29;2018:4560718. doi: 10.1155/2018/4560718 (PMC6087599; doi:10.1155/2018/4560718)
Supplement: Supplementary Materials — Supplementary file 1: an example of the application of the proposed intermediate care unit-quality measurement framework. Supplementary file 2: the used search terms. Supplementary file 3: an overview of the included studies with a short description, the used comparison, severity of illness at IMCU or ICU level, and described outcomes on all three levels. [file 4560718.f1.pdf]

Supplemental File 1. Classification of Quality Parameters of the Intermediate Care Unit – an Example

	Intermediate Care Unit - level	Intensive Care Unit - level	Hospital - level
Structure	- Human Resources (# 1 **nurse: patient ratio decreases**)	- Facilities (extra ICU beds necessary)	- Human Resources more nurses needed
Process	- Type of Admissions (workload, caseload severity decrease. Correct/incorrect admissions).<br>- Patient flows (in-and out of IMCU, re-admissions decrease)	- Type of Admissions (nursing workload, caseload severity, low-risk admissions, inappropriate admissions increase)<br>- Accessibility of ICU decreases<br>- Length of ICU Stay increases	- Length of Hospital Stay increases
Outcome	- *(Standardized) Mortality rate decreases**<br>- *Comorbidity decreases**	- (Standardized) Mortality Rate decreases<br>- comorbidity decreases	- Overall in-hospital Mortality Rate increases<br>- Costs of admission increases<br>- Patient satisfaction increases

This supplemental file shows a hypothetical example of how to interpret Figure 1 in the article. In this example, the nurse to patient ratio at the IMCU is decreased (IMCU-structure), e.g. due to hospital policy. This leads to a decreased nursing workload, caseload severity and re-admission rate at the IMCU due to the lack of possibility to admit more severe patients (IMCU-process) and thus to a decreased mortality at the IMCU (IMCU-outcome). Also, this changed process may lead to more beds necessary at the ICU (ICU-structure), less accessibility of the ICU and a lower caseload severity at the ICU (ICU-progress) and thus to a lower mortality at the ICU (ICU-outcome). Potentially also, due to less optimal allocation of available resources, the hospital –overall- needs more nurses (hospital-structure), the in-hospital length of stay increases (hospital-process) and the overall mortality and costs increase (hospital-outcome).

List of abbreviations: IMCU = Intermediate Care Unit; ICU = Intensive Care Unit.

Supplemental file 2. Search terms

(Medium Care Unit*[title/abstract] OR

Medium Medical Care Unit*[title/abstract] OR

Intermediate Care Unit*[title/abstract] OR

Intermediate Medical Care Unit*[title/abstract] OR

Medical Intermediate Care Unit*[title/abstract] OR

High Care Unit*[title/abstract] OR

High Medical Care Unit*[title/abstract] OR

High Dependency Unit*[title/abstract] OR

High-Dependency Unit*[title/abstract] OR

Progressive care unit*[title/abstract] OR

Step up/down unit*[title/abstract] OR

Step-up unit*[title/abstract] OR

Step up unit*[title/abstract] OR

Step-down unit*[title/abstract] OR

Step down unit*[title/abstract] OR

Transitional care unit*[title/abstract] OR

Medium Care Ward*[title/abstract] OR

Medium Medical Care Ward*[title/abstract] OR

Intermediate Care Ward*[title/abstract] OR

Intermediate Medical Care Ward*[title/abstract] OR

Medical Intermediate Care Ward*[title/abstract] OR

High Care Ward*[title/abstract] OR

High Medical Care Ward*[title/abstract] OR

High Dependency Ward*[title/abstract] OR

High-Dependency Ward*[title/abstract] OR

Progressive care Ward*[title/abstract] OR

Step up/down Ward*[title/abstract] OR

Step-up Ward*[title/abstract] OR

Step up Ward*[title/abstract] OR

Step-down Ward*[title/abstract] OR

Step down Ward*[title/abstract] OR

Transitional care Ward*[title/abstract])

NOT (

Child*[title/abstract] OR

Paediatric*[title/abstract] OR

Infant*[title/abstract] OR

Neonate*[title/abstract] OR

Pediatric*[title/abstract])

These terms were used in Medline, Embase & Cochrane

Supplementary file 3. Studies Included in Systematic Review

Article	Study Description (n= # of IMCU patients, unless specified otherwise)	Comparison of Interest (comparison used in study)	Severity of Illness IMCU	Severity of Illness ICU	Outcomes IMCU –level	Outcomes ICU –level	Outcomes Hospital – level
**Armstrong 2015 [16]**	IMCU cohort (n=87)	None (Day shift to evening and night-shift)	APACHE II 11 (SD 5), NAS 43.9 (SD 13.2) during day-shift		Mortality 3%		In-hospital mortality of IMCU patients 11%
**Auriant 1998 [17]**	IMCU cohort (n=433)	None	SAPS II 22.3 (SD 12.0)		Mortality 2.7%, length of stay 3.1 (SD 2.3) days		In-hospital mortality of IMCU patients 8.1%,
**Bertolini 2005 [18]**	IMCU (n=65), multi-centre (6 IMCUs)	None (ICU cohort with respect to costs)			Mortality 7.7%		
**Byrick 1993 [28]**	ICU cohort, before-after (n=570)	ICU with and without IMCU		TISS 37.1 (SD 15.9) with and 27.9 (SD 14.9) without IMCU. APACHE II 21.9 (SD 7.4) with IMCU and 18.6 (SD 7.4) without IMCU		ICU length of stay 8.5 days (SD 15.8) with and 6.7 days (SD 22.5) without IMCU	Hospital length of stay of ICU patients 37.3 days (SD 42.1) with and 26.5 (SD 31.3) days without IMCU. In-hospital mortality of ICU patients of 31.4% with and 25% without IMCU.
**Capuzzo 2014 [12]**	ICU cohort, multi-centre (167 ICUs, n=5834)	ICU with and without IMCU		ICUs with IMCU significantly higher SAPS II (37 (IQR 24-53) vs 29 (IQR 20-45)) and SAPS II (35 (IQR 23-48) vs 28 (IQR 19-41))		In hospitals with IMCU: Higher ICU- length of stay (3.7 vs 2.8 days). ICU-mortality higher (19.5% vs 16.3%)	In hospitals with IMCU: Significantly reduced adjusted in-hospital mortality (OR 0.63). Higher hospital length of stay (13.9 vs 11.0 days)
**Clarke 1996 [19]**	IMCU cohort (n=92), before-after	Nursing interventions	APACHE II 12 (SD 5.4)		Mortality 6.52%, length of stay 2.6 days		In-hospital mortality of IMCU patients
**Coggins 1996 [20]**	IMCU cohort (n=1091)	None			Mortality 1.92%, discharge to ICU 4.86%		
**Crosby 1990 [21]**	IMCU cohort (n=611)	None			Mortality 1.96%, discharge to ICU 1.64%,		Hospital length of stay of IMCU patients of 38 days

This Supplementary file shows the included studies in the Systematic Review, with a short description, the used comparison, severity of illness at IMCU or ICU level and described outcomes on all three levels. The severity of illness and outcomes at the ICU level are comparative. They compare the situation with IMCU to the situation without the IMCU. For the continuous data, either the mean or medians were reported. If the mean is reported, the SD in brackets is given here. If the median is reported, the IQR in brackets is provided.

Definition of Abbreviations: IMCU = Intermediate Care Unit; ICU = Intensive Care Unit; APACHE = Acute Physiology And Chronic Health Evaluation; SAPS = Simplified Acute Physiology Score.

Supplementary file 3. Studies Included in Systematic Review

Article	**Study Description** (n= # of IMCU patients, unless specified otherwise)	Comparison of Interest (comparison used in study)	Severity of Illness IMCU	Severity of Illness ICU	Outcomes IMCU –level	Outcomes ICU –level	Outcomes Hospital – level
**Davies 1999 [22]**	IMCU (n=300) and hospital cohort (n=9625), before-after	Introduction IMCU			Mortality 3%, Discharge to ICU 3.00%, Length of stay 1.5 (0.67-1.5) days	After introduction of IMCU: The ICU mortality significantly increased from 29% to 58%	In-hospital mortality of IMCU patients 19%. Overall in-hospital mortality 3.2%(with IMCU) compared to 2.16%,
**Dhond 1998 [29]**	IMCU (n=367) and ICU cohort (n=570), before-after	Introduction IMCU	APACHE II 9 (IQR 8 – 9)	After introduction IMCU: APACHE II remained 13 (IQR 12-15)	Mortality 3%	After introduction of IMCU: ICU mortality increased from 15% to 23%	
**Eachempati 2004 [30]**	IMCU (n=498) and ICU cohort (n=1783), before-after	Introduction IMCU	After introduction of IMCU: Significant increase in APACHE II from 13.4 (SD 0.3) to 14.2 (SD 0.3) in IMCU + ICU patients compared to ICU patients before introduction		After introduction of IMCU: Significant decrease in mortality of 8.2% to 6% in IMCU + ICU patients compared to ICU patients before introduction		
**Franklin 1988 [2]**	Hospital cohort (n=22,998)	Introduction IMCU			Mortality 2.1%	Mortality 40.1% without and 35.5% with IMCU	In-hospital mortality of all admitted patients 4.5% without versus 3.9% with IMCU
**Garfield 2000 [24]**	IMCU cohort (n=407)	None	TISS-28 23 (IQR 19-26), APACHE II 9 (IQR 6-12)		Mortality 4.2%		
**Gould 2010 [23]**	IMCU cohort (n=1145) of which case study is performed on ICU discharge predictors	None			Mortality 6.72%, discharge to ICU 6.72%		
**Kalayi 2001 [25]**	IMCU cohort (n=88)	None (IMCU-needing patients on ward)			Mortality 11%, length of stay 4 days.		

This Supplementary file shows the included studies in the Systematic Review, with a short description, the used comparison, severity of illness at IMCU or ICU level and described outcomes on all three levels. The severity of illness and outcomes at the ICU level are comparative. They compare the situation with IMCU to the situation without the IMCU. For the continuous data, either the mean or medians were reported. If the mean is reported, the SD in brackets is given here. If the median is reported, the IQR in brackets is provided.

Definition of Abbreviations: IMCU = Intermediate Care Unit; ICU = Intensive Care Unit; APACHE = Acute Physiology And Chronic Health Evaluation; SAPS = Simplified Acute Physiology Score.

Supplementary file 3. Studies Included in Systematic Review

Article	**Study Description** (n= # of IMCU patients, unless specified otherwise)	**Comparison of Interest** (comparison used in study)	Severity of Illness IMCU	Severity of Illness ICU	Outcomes IMCU –level	Outcomes ICU –level	Outcomes Hospital – level
**Keegan 2008 [27]**	ICU cohort (n=4053), before-after	Introduction open format IMCU		After introduction of IMCU: Acute Physiology score and APACHE III significantly increased, respectively, from 23.9 (SD 13.2) to 37.2 (SD 18.7) and from 34.5 (SD 15.3) to 49.6 (SD 20.8)		After introduction of IMCU: ICU mortality significantly increased from 1.14% to 7.27%. Standardized mortality rates increased from 0.68 to 1.20. Re-admission rate significantly increased from 9.0% to 17.0%	After introduction of IMCU: In-hospital mortality of ICU patients significantly increased from 2.9% to 11.9%. Standardized mortality rates increased from 0.83 to 1.24
**Lucena 2013 [7], Alegre 2015 [6]**	IMCU cohort (n=743)	ICU cohort	SAPS II 33.1 (SD 12.9), SAPS III 60.3 (SD 14.0), IMCU Severity Score (IMCUSS) 21 (SD 16.1)		Mortality 19.7%, Discharge to ICU rate of 9.96%		
**Nethra 1994 [26]**	IMCU cohort (n=412)	None (Different years)			Mortality 9.47% (last period)		
**Robertson 2011 [15]**	ICU cohort (n=2142), before-after	Introduction IMCU		After introduction of IMCU: APACHE II decreased from 19.67 to 19.37 (no data on SD or significance)		Re-admission rate decreased from 4% to 3% (not significant)	

This Supplementary file shows the included studies in the Systematic Review, with a short description, the used comparison, severity of illness at IMCU or ICU level and described outcomes on all three levels. The severity of illness and outcomes at the ICU level are comparative. They compare the situation with IMCU to the situation without the IMCU. For the continuous data, either the mean or medians were reported. If the mean is reported, the SD in brackets is given here. If the median is reported, the IQR in brackets is provided.

Definition of Abbreviations: IMCU = Intermediate Care Unit; ICU = Intensive Care Unit; APACHE = Acute Physiology And Chronic Health Evaluation; SAPS = Simplified Acute Physiology Score.

Supplementary file 3. Studies Included in Systematic Review

Article	Study Description (n= # of IMCU patients, unless specified otherwise)	Comparison of Interest (comparison used in study)	Severity of Illness IMCU	Severity of Illness ICU	Outcomes IMCU –level	Outcomes ICU –level	Outcomes Hospital – level
**Solberg 2014 [11]**	ICU cohort (n=1027), before-after	Introduction IMCU		After introduction of IMCU: APACHE II non-significantly increased from 19.3 (SD 8.1) to 19.6 (SD 7.6), TISS-28 significantly increased from 28.7 (SD 10.6) to 30.6 (SD 11.0)	Length of stay 3.4 days.	After introduction of IMCU: Mortality significantly increased from 16% to 21%. ICU readmission rate and length of stay did not change.<br><br>Inappropriate use of overflow units (PACU, cardiac ICU) all significantly decreased. The number of patients refused at the ICU did not change.	
**Yoo 2015 [14]**	IMCU cohort (n=318), before-after	Intensivist and non-intensivist IMCU staffing format			After introducing open format: no difference in adjusted mortality, ICU discharge rates (4.0% vs 7.7%) or length of stay (6.7 vs 9.0 days)		After introducing open format: no significant difference in in-hospital mortality of IMCU patients (9.1% vs 11.9%)
**Wunsch 2015 [31]**	IMCU and ICU cohort, multi-centre (192 IMCUs and ICUs, n= 64,064)	Separate versus integrated IMCU (only post-ICU patients)	APACHE II 17 (IQR 13-21) in integrated IMCUs and 16 (IQR 12-20) in separate IMCUs		Length of critical care was 4.7 (IQR 2.4-8.8) in integrated and 3.8 (IQR 1.9-8.4) in separate IMCUs		In-hospital mortality of ICU and IMCU patients 16.2% (integrated) versus 19.0% (separate).

This Supplementary file shows the included studies in the Systematic Review, with a short description, the used comparison, severity of illness at IMCU or ICU level and described outcomes on all three levels. The severity of illness and outcomes at the ICU level are comparative. They compare the situation with IMCU to the situation without the IMCU. For the continuous data, either the mean or medians were reported. If the mean is reported, the SD in brackets is given here. If the median is reported, the IQR in brackets is provided.

Definition of Abbreviations: IMCU = Intermediate Care Unit; ICU = Intensive Care Unit; APACHE = Acute Physiology And Chronic Health Evaluation; SAPS = Simplified Acute Physiology Score.
